# Supplementary material for: Just-in-time: Gaze guidance in natural behavior
Source: PLoS Comput Biol. 2024 Oct 24;20(10):e1012529. doi: 10.1371/journal.pcbi.1012529 (PMC11537419; doi:10.1371/journal.pcbi.1012529)
Supplement: S1 Table — Significant differences in the proportion of fixations on the ROIs in the HARD and EASY trials as revealed by a cluster permutation test with a Bonferroni corrected significance threshold of 0.006. (PDF) [file pcbi.1012529.s001.pdf]

Just-in-time: Gaze Guidance in Natural Behavior.

Supporting Information

Table S1. Significant differences in the proportion of fixations on the ROIs in the HARD and EASY trials as revealed by a cluster permutation test with a Bonferroni corrected significance threshold of 0.006.

ROI	time start(s)	time end(s)	duration(s)	p-value
previous target object	-2.75	-0.75	2.00	<0.001
current target object	-2.75	-2.25	0.50	<0.001
current target shelf	-2.75	-1.00	1.75	<0.001
next target object	-2.75	0.25	3.00	<0.001
next target shelf	-2.75	0.75	3.50	<0.001
other objects	-2.75	-0.75	2.00	<0.001
other shelves	-2.75	-0.75	2.00	<0.001
previous target shelf	-2.00	2.00	4.00	<0.001
current target object	-1.75	-1.25	0.50	<0.001
previous target object	-0.25	2.00	2.25	<0.001
other objects	0.00	2.00	2.00	<0.001
current target object	1.00	1.75	0.75	<0.001
other shelves	1.00	2.00	1.00	<0.001
next target object	1.25	2.00	0.75	<0.001
