# Supplementary material for: Just-in-time: Gaze guidance in natural behavior
Source: PLoS Comput Biol. 2024 Oct 24;20(10):e1012529. doi: 10.1371/journal.pcbi.1012529 (PMC11537419; doi:10.1371/journal.pcbi.1012529)
Supplement: S2 Table — Fixed effects coefficients of the linear mixed model detailing estimated latency of first fixations w.r.t the current target object during the action execution epochs. (PDF) [file pcbi.1012529.s002.pdf]

Just-in-time: Gaze Guidance in Natural Behavior.

Supporting Information

Table S2. Fixed effects coefficients of the linear mixed model detailing estimated latency of first fixations w.r.t the current target object during the action preparation epochs

Model:

$$latency \sim 1 + trial\_type * ROI\_type + (1 + trial\_type + ROI\_type|Subject)$$

	Estimate	95% CI	t	p
Previous Target Shelf	-0.36	[-0.38, -0.34]	-35.49	$<$ **0.001*****
Previous Target Object	-0.36	[-0.39, -0.34]	-29.64	$<$ **0.001*****
Other Shelves	-0.29	[-0.31, -0.27]	-32.24	$<$ **0.001*****
Other Objects	-0.28	[-0.30, -0.26]	-31.05	$<$ **0.001*****
Next Target Object	-0.08	[-0.10, -0.06]	-7.85	$<$ **0.001*****
Next Target Shelf	-0.05	[-0.08, -0.03]	-3.97	$<$ **0.001*****
Current Target Shelf	-0.04	[-0.07, -0.02]	-3.25	**0.001*****
**Interactions**				
Trial Type : Previous Target Shelf	-0.06	[-0.09, -0.03]	-3.94	$<$**0.001*****
Trial Type : Previous Target Object	-0.08	[-0.11, -0.05]	-4.62	$<$**0.001*****
Trial Type : Other Shelves	-0.09	[-0.11, -0.06]	-5.72	$<$**0.001*****
Trial Type : Other Objects	-0.10	[-0.13, -0.07]	-6.55	**0.001****
Trial Type : Next Target Object	-0.10	[-0.13, -0.07]	-6.22	**0.001*****
Trial Type : Next Target Shelf	-0.03	[-0.06, -0.00]	-1.74	0.08
Trial Type : Current Target Shelf	-0.04	[-0.07, -0.01]	-2.42	**0.016***
