# Supplementary material for: Just-in-time: Gaze guidance in natural behavior
Source: PLoS Comput Biol. 2024 Oct 24;20(10):e1012529. doi: 10.1371/journal.pcbi.1012529 (PMC11537419; doi:10.1371/journal.pcbi.1012529)
Supplement: Attachment — Submitted filename: Response to Reviewers.pdf [file pcbi.1012529.s004.pdf]

Response to Reviewers

Reviewer #1

I think this is an interesting study examining how task complexity influences gaze patterns. The task itself is very clever in that the manipulation clearly influences the complexity of the planning stage, but not necessarily of the execution. I feel confident that something interesting could be found in this dataset, but the current presentation makes it very difficult for me to see what. I do not understand why many of the analyses are done in the way they are, or even at all. This does not mean that it was wrong to do them, or to do them in this way, but I think the reasoning needs to be clarified. In combination with the fact that many of the analyses seem superfluous to me (because the outcome is trivial) the paper left me with the feeling that I did not learn anything. I think the authors should really consider what they want to demonstrate or explore with this study, and focus on that issue. Doing so will probably involve completely rewriting the paper. The main issues that I have with the current presentation are explained below (in random order).

I think the authors need to explain the task much earlier, not in a methods section at the end of the paper. Now they force the reader to look back and forth. It is fine to keep details that are not needed for understanding the results for the end of the paper, but it is currently impossible to understand the results without first looking at the various instructions that specify whether a task is simple or complex. This problem starts already in the Author Summary, where the sentence starting with “By only studying the sequence of fixations based on past, current and next actions” makes no sense at all without knowing what you mean by ‘only’ or by the three types of actions.

The introduction is extremely broad, and introduces lots of aspects, but then never really becomes concrete. This is probably partially responsible for the whole study then failing to really answer any question. The most concrete prediction is that “an abundance of look-ahead saccades would be evidence of elaborate cognitive planning” (line 95), but this is then never really examined empirically, while lots of other details are. To do so would require a more detailed study of what is now considered the ‘other’ category, especially before grasping. Now, the data in Figure 3 suggest that there is no such elaborate cognitive planning.

There are several claims that are not explained in the results section as well. For instance, on line 137: “Further, the expectation was that the subjects would move the objects randomly and not display a preference for object pickup and drop-off locations”. Why?

Surely there are some more comfortable locations or movements. Moreover, they may want to move less far (as indeed turns out to be something the authors require for movements to be 'optimal'). It is also claimed that the tendency to move objects up and to the left is not optimal, but it is not clear why. In many cases there are presumably several equivalent options. In such cases it may not be sub-optimal to select a certain one. Of course figure 2 suggests that participants wanted to order the items in a certain way in the top left, and perhaps even did so when that meant moving one (or even several) items more than would be necessary if they used different rows and columns. Apparently they did not consider leaving a central row empty. This could just be the way they interpreted the instruction, or it could feel easier. It can probably account for much, if not all, of the "sub-optimal" behavior. This is maybe interesting, but not really relevant for the question.

In general, the reasoning needs to be clearer. On lines 164-168: "The presence of fixations on the next target object even before the end of the current action execution shows that the target for the next action is queued in shortly before ending the current action." This is probably true, but cannot be seen from these data because this is the median end of action execution. You would need to synchronize on the end to really make claims about this. "Moreover, the proportion of fixations on the 'other' ROI although lower than on the current target shelf, indicates a search process for task-relevant objects/shelves." Again, maybe true (I am not sure what lower on the shelf is referring to), but impossible to judge from the bundled 'other' category. "In sum, in the action execution phase, the results show two primary functions of gaze, first to assist in the completion of the current action and second to locate or search the objects for the next action." This is certainly true, but you do not need any data for this. Such empty claims, throughout the paper (and the paper is full of them), make it impossible to see when a real finding is being reported. For instance, on the next page all sorts of trivial claims about fixations for planning are made, rather than testing some clear hypothesis about when such fixations take place, or even exploring when they take place. That any difference you see before grasping the next object might contribute to planning is obvious. What are the authors trying to explain?

The summary (from line 222) is also strange: "First, despite large behavioral differences between the two task types, the eye movements do not show corresponding large deviations". I see no justification for considering the behavioral differences to be large and those on eye movement not to be large, and certainly no justification to directly compare them in this manner, because the behavioral differences are mainly between the movements while the gaze is mainly analyzed within movements. The subsequent claims relating "to relevant cognitive schemas" and "queuing objects into the working memory for subsequent actions" and schemas not corresponding "to broader planning strategies for task completion" are so vague as to be totally meaningless in my opinion. Sentences such as "While look-ahead fixations occurred predominantly before the initiation of the

immediate action, the checking fixations occurred in parallel with the action execution" (line 384) are also completely meaningless because they are true by definition. I think the authors need to critically look through the whole paper and remove all sentences that are not actually relevant or meaningful.

The sentence on line 257 nicely exemplifies what is wrong with the statistics in this study: "In order to statistically analyze the differences in mean relative net transitions in a trial we used a linear mixed effects model". Such statistics are designed for evaluating a hypothesis, not for exploring differences. The authors are aware of the statistical problems, and conduct corrections for multiple testing to avoid the most obvious ones, but it is not at all clear to me why one would conduct such tests at all if the goal is to explore where the differences might be. Altogether, an emphasis is placed on irrelevant differences, just because they are 'significant', whereas the main finding is that they look very much the same. I think the authors need to not just dump all the possible analyses on the reader, but to explain why certain analyses are informative and present those in more detail so that it is evident how the outcome should be interpreted. There is no point dwelling on whether differences at some time interval are significant if you do not even know whether it is correct to match those time intervals (sometimes time itself is considered, and sometimes relative time during an epoch; if the timing is not the same between conditions there must be a difference in one of the two measures). Indeed, without any clear hypothesis the interpretation is meaninglessly vague: lending evidence to "differential gaze guidance behavior" (line 279). Mentioning completely unrealistic options in the argumentation also does not help: e.g. "if fixations are made haphazardly" on line 297 or the fact that the occurrence of first fixations on various items was different than that for the target object on line 339.

Why bother testing the difference between the time taken to perform the tasks with a t-test? Is it relevant whether the difference is statistically significant? My feeling is that it is obvious that there will be a difference, and the question is whether the difference is large enough for one to expect to see differences in gaze related to planning. Or do the authors really consider this to be a hypothesis that needs testing? I will stop with this example because it nicely exemplifies the need to rethink the reasoning behind the experiment, or at least to present such reasoning in a clearer and more concise manner.

On line 145 again ROIs are introduced without the reader knowing what they are. A few sentences later the reader is referred to the methods section. That really disturbs the flow of reading the paper. If you need to know this to understand the results it needs to be explained. This can be very brief: the current object and where to put it, and the object and place to put the previous and next object. The seventh ROI is the rest.

Thank you for your thorough and very helpful feedback. In an extensive revision, we have addressed your comments regarding the reasoning and language in our paper. We have rewritten the results and discussion section, and parts of the introduction to clarify the central message of our study. We have added details about the study's objectives at the end of the introduction section. We have also added introductory paragraphs in the results section, briefly describing the task, data pre-processing steps, and the different categorizations. In the results section, we have organized the statistical tests in tables and simplified the text wherever possible. We have added summary paragraphs at the end of each result section to better drive each analysis's message. We have similarly reframed the Author's Summary. This has highly improved the readability of the paper.

We also added some new analyses to clarify the central message of the paper. Specifically, we have added behavioral analysis regarding the optimal actions. In the current version, we compare human performance in each task to the performance of a depth-first search model for the same task initializations. We have further added behavioral analysis on the duration of the planning and execution periods so we can compare the corresponding gaze behavior in these epochs. In the gaze behavior analysis, we have further distinguished the ‘other’ category region of interest. We re-analyzed the data based on ‘other objects’ and ‘other shelves.’

Additional details:

Why ignore all fixations with a duration greater than 3.5 times the median absolute deviation of the population fixation duration (line 501)? Does this mean you are removing all instances in which the participant really has to attend to the object in question? Simply removing such fixations can clearly bias the outcome. What happens with the missing time in the analysis?

By rejecting fixations that were 3.5 times the median absolute deviation (MAD) of fixation duration, we removed about 2.4% of the overall fixation data that were outliers. The threshold computed by 3.5*MAD of the population fixation duration was 1.03s. Hence, we removed all fixations that were greater in duration than 1.03s. These rejected fixation samples were replaced with NaN values. As these fixations were outliers, including them in the analysis would have biased the average behavior. We have also added this explanation to the text.

Why compare the number of displacements with 5000 random possibilities, and not simply with the actual configurations? The authors could identify exactly which trials had additional movements. Were the too easy options removed from the simulations as well (as they were in the data for some reason)? If not, can that explain why the number of movements was lower in the simulations?

Addressed. Thank you for the suggestion. In the revised version of the paper, we have changed the analysis to compare the number of displacements by the participants with the optimal solutions for the shelf configurations presented to the participants in each trial.

Why are the whiskers in Figure 2 1.5 times the IQR and not the actual 95% confidence interval or something like that?

Addressed. We have simplified all plots to show the mean and the actual spread of the data to ensure irrelevant details do not detract from the message.

Reviewer #2

This manuscript presents an experiment concerning the distribution and timing of eye fixations during a new physical manipulation task. The study is well framed in the literature and provides confirmation of previous findings but also a more detailed picture of how planning and eye-hand coordination evolve during reaching, grasping and placing of objects. The methods used to verify the hypotheses appear adequate and valid and the drawn conclusions warranted. I mostly have some understanding remarks and some interpretation comments, that if discussed, might help the reader get a clearer picture.

1. I assume this is an editorial choice, rather than the authors'choice, but I found a bit annoying that methods were relegated to the end of the manuscript. The transition from the introduction to the results is quite abrupt and I had to retrieve in the last section what participants had to do to have a rough understanding of what the results were about (and also to understand most of Fig. 1). I found the description at the beginning of sec. 2 a bit short and I was left wondering how exactly participants were supposed to execute the tasks. Also, to illustrate the division into planning and execution epochs, Fig. 1D might be referred here.
In section 2.2, the 7 ROIs are suddenly mentioned as a known fact, but these were never introduced before. If no short recall can be given here at least section 4.5.2 should be referred and the legend of Fig. 3.

Done - Thank you for your suggestions. We've added a brief summary of the methods in the initial paragraphs of the Results section. This includes the pre-processing steps, the number of trials and subjects we finally analyzed, and the data segmentation and labeling method.

2. Although I understand the rationale of dividing each "move" in a pre- and post-grasp phase, I wouldn't define them as action planning and action execution epochs. Indeed, in such a task it is somewhat difficult to exactly pinpoint when decision-making and planning take place. The [-2, 2] seconds interval is reasonable but it seems arbitrary and prior to a grasp a reaching movement is issued which is already an action execution. At the same time, after grasp and during placing also planning is at work, as also shown by fixations on the next target. Hence, the temporal distinction between action execution and planning is in my view less strict than what conventionally defined here. It would be actually really interesting to investigate whether such processes (action planning and action control) could be pinpointed on the basis of the oculomotor behavior. Looking at Fig. 4 (and considering Fig.6D) it seems indeed that the different modes in the planning epochs signal different processes at play. While part of the initial fixations are still on the previous target/shelf,

probably making sure the object was placed in a stable way, another large portion is on the other region, signaling a searching and decision making behavior between different possible targets, before converging on the actual current target.

Addressed - Thank you for this feedback. In the corrected draft, we have made the time window [-3s, 2s] i.e., 3s before grasp onset and 2s after. This window approximately corresponds to the mean planning and execution duration, respectively. Furthermore, we have added further analysis in section 2.1 (Fig2 B1), which shows that task complexity significantly affected the duration of planning and not execution. This further proves that no meaningful 'planning' happens while the immediate action is in progress.

We agree with your statement that it is difficult to pinpoint when the plan to grasp the object takes place, so calling the action epochs planning might be misleading. We have changed it to action preparation to identify it as a time period before the grasp that also facilitates the action. We have also prefaced the use of the terms in the introduction.

3. Following the last comment, it could be helpful to differentiate also the other ROI between other targets and other shelves. This would give some indication of whether, for example other targets are preferentially looked at prio to the grasp (i.e., deciding which should be the current target) and whether other shelves are considered after the grasp.

Done - Thank you for this suggestion. We changed the 'other' category to 'other objects' and 'other shelves'. We can see from the results in Fig3 that the mean fixations across time are equal in both categories. Complex tasks were associated with more fixations on both 'other objects' and 'other shelves' early in the action planning phase, indicating comprehensive search behavior. On the other hand, in the execution phase, complex tasks elicited more fixations on 'other objects,' indicating these fixations were likely toward elaborate task monitoring.

4. I do not understand why broader planning strategies were expected, or a higher number of look ahead fixations, but perhaps I misundertood the reasoning. As I gather, and as partly discussed in the conclusion, this task was a) self-paced; 2) did not prescribe a strict sequence of steps. In both the easy and hard case, at any time step a target must be chosen complying with the current task, independently of future targets (e.g., if one is filling a row with red targets, it just needs to find the next red target). At most, in the hard trials, the participant needs to check (with checking fixations) that the same color or shape is not already present in the considered row/column, but they don't need to plan two or more steps ahead, rather indeed they just use the world as an outside memory.

Thank you for the comment. We have clarified the reasoning in the revised draft (line number 92). From our perspective, bereft of an “action script,” participants would implicitly search the problem space and decide on a couple of moves before implementing them (e.g. in Chess or Go where people plan some of their moves in advance). The data shows clearly that this was not the case. Instead, participants relied predominantly on visual search and fixated on the target objects just in time of the overt action.

5. Similarly, I suspect there is some possibly culture-based implicit strategy behind the detected spatial heuristics. Again, participants were not constrained in the order in which they sorted the targets but since in western countries writing goes left to right and top to bottom, participants sorting stuff might be biased to fill the shelves this way. This would also explain why usually the most right columns and the most bottom row were left out (since there were 16 objects and a 5x5 grid). This is just an intuition, I do not have pointers to the literature in this sense. Since the task is self-paced, no optimality is enforced or rewarded (e.g., to minimize the displacements) and such a left to right/top to bottom strategy would simplify the step to step planning by offloading the decision to the spatial structure of the grid and the usual scanning strategy.

Done - Thank you for the comment. The volunteers in our study were predominantly students who could read and speak in English or German. It would be interesting to investigate the cultural differences in information gathering behavior. Indeed, there is some evidence from studying the gaze behavior of bilinguals that a horizontal bias exists and is influenced by the reading direction (Afsari et al. 2016). As we did not reward or penalize any specific action choice, the observed sorting behavior is surprising and a purely emergent behavior related to the experimental task. Such a prominent spatial heuristic is further evidence of using the world as an external memory and offloading cognitive work on the environment.

Afsari, Z., Ossandón, J. P., & König, P. (2016). The dynamic effect of reading direction habit on spatial asymmetry of image perception. Journal of Vision, 16(11), 8. https://doi.org/10.1167/16.11.8

6. The results section is interesting but really lengthy, perhaps less relevant results could be condensed.

Done - Thank you for this feedback. We have organized the modeling results in tables and refer to the significant results in the text.

Minor comments:

-how are the maps in Fig 2 C and D computed? its the overall frequency of displacements across participants?

Done - Yes. We first calculated the counts of the displacements for each pickup and dropoff location. We normalized the counts with the total number of displacements in the easy (fig2C) or hard (fig2D) tasks. The normalized counts in the figures add up to 1. This explanation is included in the revised version of the manuscript.

- line 164: as pointed out above, before and more than an increase on the next target object there's an increase in the other ROI fixations, again this might signal a look-ahead behavior in selecting the next target before ending the grasp (but yet it would need differentaiting between other target rois and other shelves rois)

Done - We have relabeled the 'other' category to 'other objects' and 'other shelves'.

-line 228-229: again, I'd say that both task types do not require to plan further ahead than the next step
Done - We have rewritten these paragraphs.

-line 265-266: this could be evidence that indeed the distinction between planning and execution epochs in an artificial one and the two processes are more intertwined

Our analysis of the planning and execution durations (Fig2 B1, B2, and Table 1) shows that planning epochs were longer than execution epochs, and the task complexity significantly enhanced this difference. Further, the execution epochs of both EASY and HARD trials were comparable in length. Although there is no significant difference in the net transitions between the two epochs, it can be inferred that gaze transitions are equally symmetric between ROI pairs in both planning and execution epochs but serve different purposes. The significant interaction between the trial type and epoch type in Table 4 further shows the decrease in net transitions primarily in the planning epochs of HARD trials. Taken together, the decrease in net transitions in the planning epochs with increased task complexity is an indication of greater search behavior. As a similar decrease in the execution epochs is not observed, the action monitoring fixations remain relatively unaffected by task complexity.

-line 19-20 it should read "Studying eye movements in moving subjects..."
-line 23: "Kollmorgen et al (2010) demonstrated THAT...."
-line 40 would be best reformulated like "Moreover, Land and Hayhoe(2001) have broadly categorized fixations while performing habitual tasks into four functional groups."
-lines 86 + 88: I'd say realistic environments rather than natural...it's still a human-made environment

-line 356: it should read "during action planning" instead of while

Done - Thank you for these corrections. We have added them to the text

Reviewer #3

This paper is an interesting piece of work that highlights with more systematic experimentation some of the early gaze control work by Hayhoe/Ballard in the 90s-00s, using a nicely-structured virtual reality task. The fact that the task is not overlearned is I agree an important point. I would have maybe also liked a small paragraph or two discussing the translatability of these findings in a VR setup to a similar experimental setup in the real world.

Thank you for your comments, we have added a paragraph at the end highlighting some pitfalls of conducting research in VR and the translatability of our work to real-world paradigms.

- The opening sentence I think maybe needs a bit of revision, I'm not sure what 'in the pragmatic turn' means in this context. I very much agree with the idea about moving from embodiment to cognition, the arguments for are quite strong.

Thank you for the comment. We have added the context for the pragmatic turn. This term alludes to the title of a previous review paper that we also cite in the present manuscript. From the perspective of embodied cognition, the pragmatic turn refers to the recent push to study the cognitive process of proactive and voluntary actions.

- Only lists refresh rate of the HMD, not of the Tobii eye-trackers in the paper as far as I can see (the frequency from the manufacturer specs appears to be 120Hz, with 0.5-1.1 degree accuracy)

Thank you for the suggestion. We have added the eye-tracker accuracy and sampling rate to the methods section.

For Figure 2, it looks like the plots for 2B have changed slightly between the preprint and this submitted version, in terms of the average values and error bars - is this due to more trials being performed, or a re-analysis of the existing data?

In the preprint, we analyzed the whole dataset without proper outlier removal. The preprint analysis is based on all trials from 55 participants. In the current manuscript, we added another preprocessing step where we removed trials where the number of object displacements was greater than 1.5 times the Interquartile Range (IQR) or less than 3. Hence, in the current version of the analysis, we have 999 trials from 48 participants.

For Figure 3, it's not very clear what the main scientific point is with respect to the differences between the horizontal traces - what point is the reader meant to take away from these in particular? It would be good to have a little box highlighting the region that is 'expanded' in the latter section of Figure 3, it wasn't very clear on initial viewing. Why also was 2 seconds the size of the window selected, was this arbitrary or based off a precedent from other work?

The time window in Fig3 now approximately corresponds to the average duration of the planning and execution, i.e., -3s before grasp onset and 2s after grasp onset. This period sufficiently describes the average fixations on the task-relevant regions during the planning and execution of actions.

The horizontal traces in Fig 3 correspond to time windows where the proportion of fixations on the same colored ROI significantly differed in the easy and hard trials. Although task complexity did not change the overall proportion of fixations on the different targets, it did affect the proportion of fixations at specific time points. These differences further help us understand the timing of gaze allocation based off on a cognitive schema.

To make the point more succinct, we have removed the expanded image of the small differences. We have also condensed the corresponding text in the results section.

For Figure 4, 'Region of Interests' should probably read 'Regions of Interest'. Line 294 should probably read 'median time to first fixation, rather than 'median time to first fixate on', but on the whole otherwise it's a good figure.

Thank you for pointing out the mistake. We have made the required corrections to the figure and the text.

Overall, in my opinion this paper would be a good fit for PLOS Computational Biology, and overall I consider it well-formed and a good study that begins to answer some outstanding questions in our field.
