# Supplementary material for: Just-in-time: Gaze guidance in natural behavior
Source: PLoS Comput Biol. 2024 Oct 24;20(10):e1012529. doi: 10.1371/journal.pcbi.1012529 (PMC11537419; doi:10.1371/journal.pcbi.1012529)
Supplement: Attachment — Submitted filename: Response to Reviewers - II.pdf [file pcbi.1012529.s005.pdf]

Response to Reviewers

Reviewer #1:

The revision has certainly improved the paper considerably, but I think the presentation could be even better in a couple of places. In my opinion, rather trivial findings are sometimes presented in too much detail, while important issues are hardly discussed. An example of the latter is that I wonder whether the conclusion that the study demonstrates that gaze is controlled in a just-in-time manner (lines 534-535) is justified. The term just-in-time suggests that gaze is directed to objects just before the planned arm movement starts, but actually the converse could also be true, that the hand only starts moving when gaze has located the target. Indeed, in the abstract the authors seem to be proposing the latter interpretation. I think the authors should somewhere specify precisely what they mean, because now 'just-in-time' seems to be used in both senses (which is also fine, I guess, but then it should be specified that no distinction is made between the two).

Thank you a lot for the constructive criticism and positive evaluation. In order to address all issues, we have performed additional analysis, updated figures, and revised the text substantially. Specifically, we analysed the effect of additional constraints (see below), made terminology more precise and consistent, streamlined the line of argumentation, added a discussion of critical aspects, and expanded some parts at the expense of others. We did not completely cut parts that might be perceived as obvious, as the article addresses a diverse readership, aspects might be more obvious to some but not to others. We hope this is okay with the reviewer. All major changes are marked in red. Many small changes and corrections we left unmarked for better readability.

Another issue is the comparison with optimal behaviour. Since this appears to be an important aspect of the study, I wonder whether it would not be worthwhile expanding the analysis a bit by also considering a comparison with optimal behaviour under the constraints that there should be no empty row or column between filled ones, and/or that the final configuration should be in the top left (so leaving the rightmost column and bottom row empty). Is this enough to explain performance not being optimal? I think this would be interesting to know, irrespective of the outcome. It would show how good an overview the participants have of the whole situation. Below I will propose yet another option.

Done. We did another analysis by constraining the model. It was penalized for moving objects to the last row and column. The performance of the constrained model does not significantly differ from that of the unconstrained model. Upon further inspection of the subjects' data, we found subjects manipulated and displaced objects from left to right and top to bottom of the shelf (Fig

2F and 2F). This behavior explains much of the sub-optimality observed in the behavior. We have added a paragraph discussing this in the results section.

I really like Figure 3, but I feel that the emphasis in the text is on the differences between hard and easy trials, but the most striking aspect to me is the similarity. The differences are easy to explain, and yet they are presented as interesting findings. We know from Figure 2B1 that preparation takes 2s in easy trials but 4s in hard trials. So if participants are looking at the current object until and just after drop-off time (right side of Figure 3) irrespective of the trial type, it is evident that this will be seen as more gaze at the previous target object about 2s before (the next) grasp onset on easy trials (which on average is the same moment) but not on hard trials (where the same moment is much earlier). Similarly, looking more at other objects for hard trials is logical because the next target you look at is more likely to be 'wrong' (and therefore 'other') if there are more constraints. I think all this is interesting, but it would be more useful to explain some of these things than to test (and reject) the completely unrealistic hypothesis that there would be no difference.

Done. Thank you for the comment. We've simplified Figure 3 and the accompanying text to emphasize the similarity in the gaze patterns.

Minor issues

The statement "The average fixations aligned to the action onset revealed gaze guidance was driven just in time" on line 451-452 ignores the possibility that the action starts as soon as the target is found. Thus, gaze is not just in time but the action starts when the target is found.

Done. We have added a paragraph to the discussion section describing what we mean by just-in-time.

Is the fact that a depth-first search model was used to find the optimal behaviour relevant? Was the search done to a limited depth? Otherwise I would remove the detail or explain why this particular search method was best here. Providing irrelevant details distracts the reader. I started wondering why this particular method was used, when probably it was just an arbitrary choice because it should not matter what kind of search is used.

Done. Your comment gives a precise description, and we have removed the 'depth-first' model specification.

The authors did not understand my concern with removing 'outliers'. My concern is not about the method, but about whether one should really consider them to be outliers. By doing so the authors are assuming that participants should always be doing the same thing, with random variability, but there may be a reason to sometimes fixate a certain object for a long time. It was just a thought. The authors do not need to do anything about this if they do not want to, but they should realise the assumption that they are making.

The task (line 114) does not make sense before the reader knows what the objects are, so that needs to be added at the beginning of the results section. Only when you know that there are 4 different shapes, each in 4 different colours, does the HARD instruction make sense.

Done. We've added the details of the number of objects on the shelf and their specifications.

On line 124 I could not figure out what 'gaze position and direction data' means. From the methods it becomes apparent that gaze direction was used to determine angular velocity. Maybe remove or explain 'position'.

Done. We have removed "eye position".

I wonder about the analyses that include index (Figure 2B2). We are shown a fit line, without data points. In this case, I think showing the data points might be informative. It makes sense for the preparation to take more time for hard trials, but I would have expected a different slope. The last point(s) could have been fast, because there are few options left so it should be easier to decide. The fact that the first movement was fast corresponds well with the fact that participants did not optimise their choice of row and column but simply used the top left part of the shelves. If participants do not minimise the number of movements they might just first randomly place appropriate objects on the first row, ignoring conflicts with objects already present on other rows, in which case it makes sense for the first row to be fastest. I think adding the values as faint dots (as in B1) or at least adding the means for each grasp index might be informative as to such strategies. The latter strategy could even also be simulated to see whether the actual performance is consistent with this. I think analysing the data in more detail might be much more informative than the current statistical approach. It is evident from the figure that participants are not optimal, but testing the null hypothesis is very tricky, because they cannot be better than optimal so any test based on normal distributions cannot really be valid. I think the results are very clear (e.g. figure 2C) but the statistical tests leave me with the impression that I am being fooled. And this while the data themselves are so interesting, and probably could even reveal more about how the task is done.

Addressed. We perfectly agree with your line of reasoning. We have added data points to figure 2B2 and shortly explained these aspects. Further, we have performed and documented additional analysis as described below.

I think the authors could specify in lines 208-210 how specifically subjects "offset their cognitive load" and what kind of "sub-optimal strategy" they could have adopted. I have made some suggestions above (pick rows and columns in advance; ignore conflicts with items on future rows). I think a combination of looking at the preparation times and at the placing orders might actually reveal how participants do this.

Done. We have added an analysis, as shown in figures 2F and 2G, which shows participants manipulated and displaced objects from left to right and from top to bottom of the shelf as the trials progressed. This strategy explains the lower preparation duration at the beginning of a trial and the longer duration towards the latter part.

An issue that was not quite clear to me was whether the subjects were instructed to make as few moves as possible or to be as fast as possible. Maybe this should explicitly be mentioned, even if no such instruction was given, because 'optimality' is defined by the authors in terms of minimising the number of moves, but maybe the participant can be faster if he or she does not worry about making additional moves (saving preparation time; maybe this is what the authors have in mind when they mention reducing cognitive load, although there may be other reasons to reduce cognitive load than reducing the required time).

Done. We have clarified in the text that subjects were not given any further instruction on how to complete the task and were not constrained by time.

In my opinion the analysis of the gaze transition behaviour is superfluous and could be removed. It is a clever analysis, but the outcome is trivial. Of course the transitions will be more variable for the hard task. This is related to looking more at other objects. At least this should be discussed rather than this analysis being presented as something independent. But doing an analysis and then discussing that the results are trivial seems like a waste of time and space to me.

Addressed. This analysis is important as it emphasizes a circularity in the fixation behavior where subjects exhibit more return fixations to already looked-at objects. It also shows that subjects' scant use of working memory by not encoding already fixated objects into memory. In contrast, an optimal use of working memory would require fewer refixations and show more forward fixations. We have streamlined the presentation of this aspect and tried to communicate the significance better.

The most obviously superfluous analysis, in my opinion, is that described in section 2.4. Figure 5 shows the order of fixations in a clear manner. It took me a long time to figure out what the statistical analysis means and shows, just to discover that the conclusion is best supported by figure 5 rather than by all the statistics and numbers given in the text. Moreover, the conclusion on line 431 is trivial. It could not have been the other way around.

Discussed. We respectfully disagree here. A case could be made that subjects pick up an object and then search for an appropriate place on the shelf. Such behavior would lead to considerably fewer searching fixations in the action preparation periods, and the latency of first fixations would also differ significantly. The data clearly do not show such a behavior. We have tried to improve on the presentation to communicate this view.

The first part of section 5.4.1 seems to me to be a detail of the way in which the data were provided by the eye tracker. Apparently the eye tracker gives the direction as a unit vector rather than as two angles or as the position on the screens, but why bother the reader with this? It just makes the paper more complicated.

As we have made the raw data available on OSF, it would be beneficial for the reader to understand how the data was preprocessed and transformed. Furthermore, it would help in reproducing the analysis.

The statement on line 637 that the 'other' items were 'irrelevant to the action sequence' is strange. To perform optimally you may need to look at them to determine that they are not the correct item. The authors appear to only consider gaze for manipulating objects, not for finding them.

Done. We have rephrased the sentence.

On line 650 the authors mention 'novelty', but what this means is only explained later.

Done.

There is a typo on line 116: unique shape (not shapes)

Fixed.

Reviewer #2:

I thank you the authors for the substantial work in addressing the reviewers' comments. I think the manuscript has improved.

I just have minor comments:

- in many places 'proactive actions' are mentioned but I'm not sure what is meant by that: proactive to what? The manual actions do execute the plan. The gaze control, possibly, is proactive, retrieving information in advance and allowing actions to be executed.

Thank you for the comment. We have clarified in the introduction that by proactive actions we mean “willed/voluntary actions” that are not cued externally.

- I would reccomend a thorough proof-reading because of some typos and awkward formulations, e.g.:
pag 3, line 116: it should read unique shape, not shapes
pag 4, fig, 1.B caption: "The objects were randomly presented
on a 5x5 shelf at the beginning of each trial and were cued to sort objects by shape and color." I suspect what is meant is that participants were cued to sort objects?
pag 5, line 126:" we classified fixations to gaze samples...", perhaps you classified as fixation a cluster of gaze samples with duration larger than 100 ms?

Thank you for the notes. We have corrected the typos.

Reviewer #4:

The authors conducted a VR study in which they looked at gaze patterns depending on task complexity. The paradigm used is new to me and sounds like a promising approach to investigate the question. In my opinion, there is a successful descriptive analysis of the data. The study is also a good example of how we can use VR studies to investigate eye movements (or actions in general).

Thanks a lot for the positive feedback.

General Comment:
The introduction initially gave me the impression that a new model was being presented. It is said that the Hayhoe/Ballard model (which is also presented in Figure 1A) is being generalized. For me, this generalization or extension does not really exist. I cannot find an extended scheme or a new model. Rather, there are descriptive results that are analyzed and classified. Maybe it would help to create another illustration like 1A, where exactly the innovation lies? As I said in my initial comment, I think the study is definitely suitable for publication, but the scope should be more clearly defined. It is a new paradigm for investigating the planning of gaze sequences, but in my view it is not a new or generalised framework for describing them. Where is the classification of existing computational models of eye movements in planning? (e.g. https://www.nature.com/articles/s41598-018-37536-0). In my opinion (if the publication is not supposed to change much, such as a development of a computational model), there should be a clearer delimitation of the contribution, namely that it is a new paradigm and a first experimental investigation and not a new framework.

Done. We have changed the wording to 'test the Hayhoe/Ballard model'. We have also added a paragraph in the introduction that outlines the current computation models of eye movements in visual search and decision-making.

Additional minor points:

- The linked repository should be updated. The status is still from before the revisions.

Done. We have published all the data and accompanying code at https://osf.io/gq6aj/ for easy access.

- Figure 1C could be moved to the methods section. The first figure should give a general overview of the paper, the study and the motivation. For me, such technical details have no place here. Maybe throw them out and move Panel B to this position?

Done. Thank you for the pointer.

- Table 1 is incorrectly placed. It is on page 5 but is referenced on page 7. In between is Figure 2.

Done.

- Why is Figure 2 not arranged regularly? It is visually very confusing that it does not go from left to right and from top to bottom.

Done. The figure is now arranged properly.

- The conclusions regarding the analyses in Figure 2D and E are not far-reaching enough for me. A clear effect is observed and reported here. However, the subsequent classification is rather brief. It is still unclear to me why there is this spatial bias here and, above all, why it looks the way it does. You should go into this in more detail.

Done. We have added two figures Fig 2F and 2G, which explain further how the subjects manipulated objects from left-to-right and top-to-bottom. Also, we have added a paragraph discussing this phenomenon further in the discussion section.

- Where can I find details on the depth search model? I couldn't find any in the paper or in the repository. Also, would it be an idea to compare whether humans also perform depth search (or breadth-first search)?

Done. We have added the greedy search model details in the methods section and removed the depth-first model specification, which detracts from the main message. The model is depth-first because it traverses the solution tree one branch at a time, and the branch with the fewest nodes is selected as the optimal solution.

- Figure 3 overwhelms me visually. I can't extract any information from it. The line plot is already difficult, but together with the horizontal bars I can't see through it at all. Can you perhaps break this down a bit more?

Done. Thank you for the feedback. We've simplified the plot to make it more readable.

- Line 517 and following: I wouldn't necessarily classify VR as an absolute new paradigm. The paragraph almost reads as if there is hardly any existing work on it and that it has only been in use for 1.2 years. I would tone it down a bit and add more relevant literature.

Thank you for the note. We have included more references to show the use of VR for different experiments in the last two decades.
